# The Safety and Efficacy of Two-site Phacotrabeculectomy with Mitomycin C under Retrobulbar and Topical Anesthesia

**DOI:** 10.5005/jp-journals-10008-1196

**Published:** 2016-05-12

**Authors:** Raquel Rodriguez, Rachel Alburquerque, Tripper Sauer, Juan Francisco Batlle

**Affiliations:** Medical Director, Department of Glaucoma, Oftalmologia, Santiago, Dominican Republic; Member, Department of Glaucoma, Elias Santana Hospital, Santo Domingo, Dominican Republic; Resident, Department of Ophthalmology, Elias Santana Hospital and Centro Laser, Santo Domingo, Dominican Republic; Medical Director, Department of Ophthalmology, Elias Santana Hospital and Centro Laser, Santo Domingo, Dominican Republic

**Keywords:** Anesthesia, Mitomycin C, Phacotrabeculectomy, Topical, Trabeculectomy.

## Abstract

**Purpose:** To evaluate and compare the safety and efficacy of combined two-site phacoemulsification and trabeculectomy surgery with mitomycin C (MMC) in glaucoma-cataract patients with retrobulbar or topical anesthesia.

**Patients and methods:** A retrospective, nonrandomized review of consecutive phacotrabeculectomy patients with a minimum follow-up time of 6 months, no previous glaucoma surgeries, and a preoperative visual acuity (VA) greater than light perception. The main outcome measures were preoperative and postoperative VA, intraocular pressure (IOP), use of glaucoma medications, and complications. A complete surgical success required an IOP from 6 to 18 mm Hg, no visually devastating complications, no return to surgery, and no use of glaucoma medications. Qualified success allowed the use of up to two glaucoma medications. Anesthesia groups were compared by student t-tests and log rank comparison of Kaplan-Meier survival rates.

**Results:** Eighty-seven eyes (83 patients) met inclusion criteria, with a mean follow-up of 19 ± 12 months (6-57 months). The average eye gained 3.1 ± 4.9 lines of VA, lost 4.0 ± 7.1 mm Hg of IOP, and decreased 1.0 ± 1.3 glaucoma medications. Retrobulbar and topical anesthesia groups had statistically equivalent mean changes in VA (p = 0.910), IOP (p = 0.268), and use of glaucoma medications (p = 0.964). Postoperative complication rates were also statistically similar (p = 0.580). Complete success was greater in the retrobulbar group (p = 0.006), however, qualified success was equivalent in both groups (p = 0.769).

**Conclusion:** Two-site phacotrabeculectomy with MMC in West Indian patients is as safe and effective for glaucoma-cataract patients with topical anesthesia as it is under retrobulbar anesthesia, and without short-term losses in VA and the chance of serious complications from injection.

**How to cite this article:** Rodriguez R, Alburquerque R, Sauer T, Batlle JF. The Safety and Efficacy of Two-site Phacotrabeculectomy with Mitomycin C under Retrobulbar and Topical Anesthesia. J Curr Glaucoma Pract 2016;10(1):7-12.

## INTRODUCTION

Glaucoma patients with visually significant cataracts are a frequent occurrence in the aging adult population. Dual treatment has been realized for many years by combining trabeculectomy with extracapsular cataract extraction (ECCE) or phacoemulsification.^1^ However, ECCE surgery can traumatize the conjunctiva, making it less receptive to trabeculectomy. Combined surgeries have thus, become more effective and popular with the development and advancement of phacoemulsification.^2,3^ For instance, at the Elias Santana Hospital in Santo Domingo, the largest volume ophthalmology clinic in the Dominican Republic, the annual number of combined cataract-glaucoma surgeries rose 500% the year after phacoemulsification replaced ECCE as the dominant cataract extraction technique (unpublished data).^4^

The benefits of the combined phacotrabeculectomy surgery over staged cataract and glaucoma surgeries include minimizing surgical visits, ocular trauma, intersurgical IOP instability, and the overall period of visual rehabilitation. Although cataract surgery alone can reduce IOP and glaucoma medication for over 2 years, Friedman et al found combined surgery generally achieves an additional 3 to 4 mm Hg lowering.^5,6^ Trabeculectomy has been argued to have greater IOP control than phacotrabeculectomy; however, recent studies have found them equally effective.^7,8^ Other points of contention in combined surgery have been the use of antimetabolite mitomycin C (MMC) and the separation of surgical sites. A 2005 review recommended the separation of surgical sites and the use MMC for IOP reduction in advanced or progressive glaucoma patients with incipient cataracts.^7^ However, several authors have recently achieved comparable success rates with single surgical sites and without MMC.^9-11^ Patients with risks for filtration failure, such as black race, diabetes mellitus, and use of more than two preoperative glaucoma medications, are often recommended simultaneous MMC to prevent failure and glaucoma progression.^12^ However, its use is associated with increased bleb leaks and the long-term risk of blepharitis, endophthalmitis, and hypotony.^13^

Another important surgical decision is the type of anesthesia used. Topical and retrobulbar anesthesia are reported to be equally comfortable for the patient during phacotrabeculectomy.^14-17^ Although topical patients had more inadvertent eye movements, in one study, this did not pose a problem for the surgeon. Retrobulbar anesthesia, however, had a greater rate of chemosis, subconjunctival hemorrhage, and eyelid hematoma.^15^ In injection anesthesia there is always the potential for serious complications of the optic nerve including neuropathy, the so-called ‘snuff-out’ of advanced glaucoma eyes, and even death. Results of phacotrabeculectomy with topical anesthesia have been followed for a period of up to 3 months, but no long-term comparison of topical and retrobulbar anesthesia has been published to our knowledge.^18^ In this study, we have set out to evaluate the safety and efficacy of two-site phacotrabeculectomy with MMC under both retrobulbar and topical anesthesia through a retrospective review of consecutive phacotrabeculectomy cases.

## PATIENTS AND METHODS

A retrospective, nonrandomized case review was conducted on consecutive phacotrabeculectomy surgeries by a single surgeon Juan Batlle (JB) and surgical assistant Rachel Alburquerque (RA) performed at Centro Laser, Santo Domingo, Dominican Republic, between January 2000 and 2006. Phacotrabeculectomy was the recommended surgery for glaucoma patients with cataracts that impeded daily activity. This included patients with uncontrollable IOP, patients with controlled IOP, but with advanced damage to the optic nerve or visual field, and patients who could not tolerate glaucoma medications. Patients were excluded from review if they had a history of previous glaucoma surgeries, less than 6 months of follow-up, or, for statistical purposes, had an unquantifiable preoperative visual acuity.

### Surgical Technique

The procedure performed was a standard two port phacoemulsification with posterior chamber IOL implantation through a temporal wound with a trabeculectomy procedure at 12 o’clock combined with a 3-minute application of mitomycin C. Preoperatively, the pupil dilated with 1% tropicamide and 10% neosynephrine during 15 minutes. For cases done under topical anesthesia, 2% lydocaine gel was applied to the fornix. For cases with retrobulbar block, an injection of 4 to 6 cc of lydocaine followed by decompression of the globe with a Honan balloon. The eye was prepped with 5% betadine solution, the skin dried and a plastic adhesive drape applied. The lid speculum was introduced with careful eversion of the lashes under the plastic lining of the sterile drape. An 8 mm fornix-based limbal conjunctival incision was performed superiorly. The subconjunctival bleeders were cauterized with bipolar diathermy. A 4 mm scleral flap was created with a 75 beaver blade and the dissection carried to the corneal Apex with a 69 beaver blade without penetration into the anterior chamber. Three merocel sponges soaked in 0.5% mitomycin C were directed into the subconjunctival pocket and left in place for 3 minutes. The sponges were then removed and the eye copiously irrigated with balanced salt solution (BSS) to eliminate any residual mitomycin C. The microscope was then rotated to carry out the routine phacoemulsification procedure through a clear corneal incision. The corneal incision was closed with one 10-0 nylon suture and the pupil constricted with acetylcholine. The microscope was then rotated back to the trabeculectomy site where penetration into the anterior chamber was achieved with the keratome. Using a Kelly punch, a 0.5 mm posterior lip sclerectomy was performed followed by an iridectomy created with Vannas scissors. The scleral flap was closed with two interrupted 10-0 nylon sutures and the conjunctiva closed with careful attention to the cardinal sutures on each side of the scleral flap to guarantee a perfect seal of the bleb overlying the flap. The bleb and wounds were tested with a BSS cannula to confirm a watertight seal of the anterior chamber and formation of the bleb. Topical antibiotics and steroid drops were applied and the eye covered with a protective shield.

### Data Analysis

The preoperative data reviewed for each patient included age, sex, ocular history, pre-existing medical conditions, laterality, anesthesia used during surgery, IOP, VA, and number of glaucoma medications. Preoperative measures were taken from the last pre-surgical consultation. Postoperatively, IOP, VA, number of glaucoma medications, and complications were sampled from consultations nearest to 1 day, 1 week, 1 month, 3 months, 6 months, 12 months, 18 months, 2 years, 3 years, and 4 years after surgery. All visual acuities were converted to LogMAR from Snellen form with counting fingers and hand movement equivalent to 1.5 and 2.0, respectively.

Success was determined at the final consultation, except for complications that were judged failures at the most immediate consultation. Complete success was defined as an IOP no less than 6 mm Hg and no greater than 18 mm Hg, no use of glaucoma medications, no devastating visual complications, and no complications requiring additional surgery. Qualified success allowed the use of up to two medications. Kaplan-Meier survival curves were generated to evaluate success over time and to compare case variables, including gender, eye, and anesthesia, by log-rank tests. Preoperative and postoperative measures of VA, IOP, medications, and complications were compared between retrobulbar and topical anesthesia groups by two-sided student t-tests. Statistical analysis was performed with XLStat 7.0 (XLStat 2006.3, Addinsoft, New York, USA) and JMP IN 5.1 (SAS Institute, North Carolina, USA). Probability values of < 0.05 were considered significant for all analyses.

## RESULTS

A total of 87 eyes, 76 patients, who received two-site phacotrabeculectomy with MMC, met the inclusion criteria. Sixteen cases were excluded: 13 cases had < 6 months of follow-up, two cases had preoperative visual acuities no better than light perception, and one case had a previous glaucoma surgery. Follow-up ranged from 6 months to 4.9 years, with a mean of 19.0 ± 12.2 months. The total patient population was 51 (58.6%) female, 36 (41.4%) male, and the operative eye was 43 (49.4%) right and 44 (50.6%) left. Average patient age was 73.3 ± 11.5 years and 87.4% (76) of the population was greater than 60 years of age. Ethnicities were not recorded, but nearly all patients were residents of the Dominican Republic and of West Indian ethnicity.

**Graph 1:**
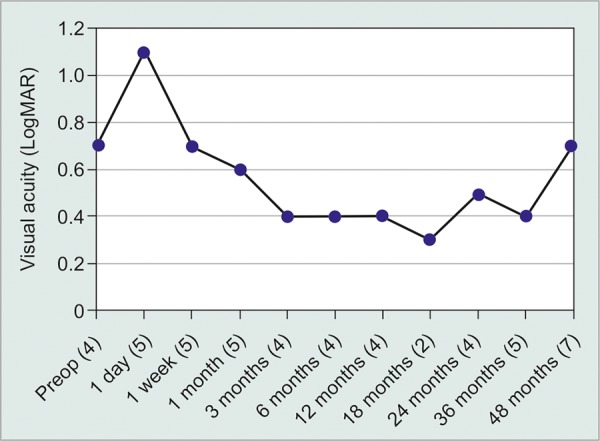
Mean LogMAR visual acuity improved over time until the later appointments as patient numbers decreased. Standard deviations in lines of acuity are noted in parenthesis

**Graph 2:**
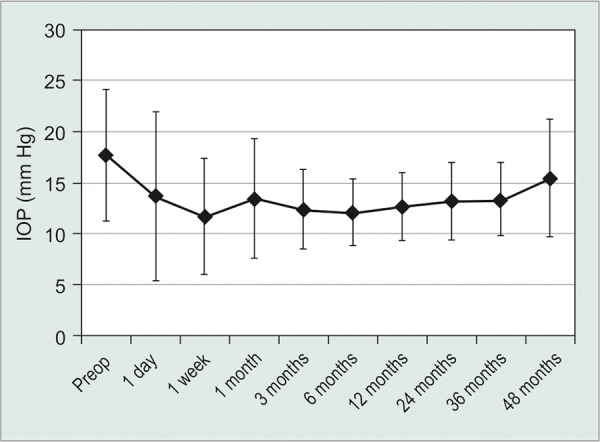
Mean IOP of all patients was steady over time. Horizontal bars represent standard deviations

**Graph 3:**
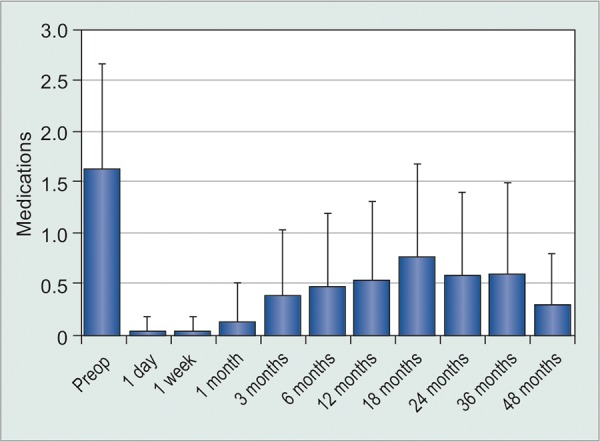
Mean number of glaucoma medications over time. No patient used medication during the first week. Horizontal bars represent the upper standard deviation

Mean preoperative visual acuity was 20/108 with a standard deviation of 4 lines. Visual acuity worsened immediately after surgery, but returned to preoperative levels 1 week after surgery as shown in Graph 1. By the most recent consultation, average VA was 20/57 ± 5 lines, 77.0% of cases had improved VA, and 10.3% of cases had diminished VA. Preoperative IOP ranged from 10 to 40 mm Hg with a mean of 17.6 ± 6.4 mm Hg. One year post-surgery, mean IOP was 12.4 ± 3.1 mm Hg, a reduction of 5.3 ± 6.8 mm Hg (Graph 2). By the final consultation mean IOP had increased slightly to 13.6 ± 4.0 mm Hg. Hypertensive and hypotensive eyes were recorded more frequently immediately after surgery as shown in Table 1. Glaucoma medications were used by 86.5 % of patients preoperatively with an average 1.6 ± 1.0 medications (Graph 3). One year after surgery, 38.5% of patients were taking glaucoma medication and no patients required more than two different medications. At the most recent consultation, medication usage was 0.6 ± 0.9, a mean reduction of 1.0 ± 1.3 medications.

**Graph 4:**
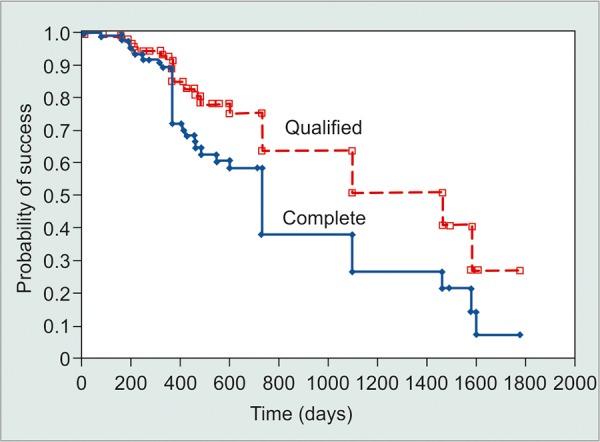
Kaplan-Meier survival curves of complete and qualified success for all patients. Paths were similar, but significantly different (p = 0.008, Log-rank test). Circles denote censored cases, signifying they were successful at the most recent consultation

Complications requiring a return to surgery occurred in four (4.6%) cases: Two cases of captured intraocular lens (IOL), one patient required additional glaucoma surgery (Ahmed valve), and one patient underwent a pars plana vitrectomy 6 months after surgery. Additional complications were single cases of blepharitis, 13 months after surgery, retinal vein occlusion, 3 months after surgery, band keratopathy, 2 months after surgery, and significant vision loss (counting fingers to light perception), 2 years after surgery. Complete success and qualified success survival rates after 1 to 4 years were 76 and 95%, 47 and 86%, 39 and 78%, and 32 and 65% by Kaplan-Meier analysis, respectively (Graph 4). Log-rank tests of qualified success rates, found no significant difference regarding laterality (p = 0.987), but a significantly higher survival rate for females than males (p = 0.025).

**Table 1:** **Table 1:** Hypertensive (> 21 mm Hg) and hypotensive (< 6 mm Hg) cases over time

		*1 day*		*1 week*		*1 month*		*3 months*		*6 months*		*12 months*		*18 months*		*24 months*		*36 months*		*48 months*	
IOP < 6		12 (14%)		6 (7%)		2 (2%)		3 (4%)		2 (2%)		3 (4%)		0 (0%)		1 (4%)		0 (0%)		0 (0%)	
IOP > 21		13 (15%)		3 (3%)		8 (9%)		2 (3%)		0 (0%)		0 (0%)		1 (6%)		1 (4%)		0 (0%)		0 (0%)	

**Graph 5:**
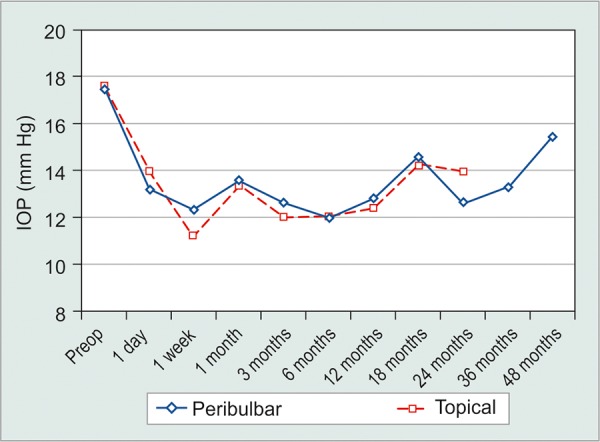
Mean IOP comparison of retrobulbar and topical anesthesia groups. Follow-up of topical cases ended after 2 years

Retrobulbar anesthesia was used in all 32 (36.8%) cases before January 1, 2003 and topical anesthesia in all cases afterward, 55 (63.2%). The retrobulbar group was 75% (24) female and the operative eye was 46.9% (15) right. Topical cases were 49.1% (27) female, 50.9% (28) right eye. Respective mean ages at operation were 73.6 ± 10.5 years and 73.1 ± 12.2 years (p = 0.838) for retrobulbar and topical cases, respectively. Mean follow-up times were 826 ± 477 days and 432 ± 171 days (p < 0.0001). Preoperatively, the retrobulbar and topical groups had statistically equivalent visual acuities [20/125 ± 5 lines and 20/112 ± 5 lines (p = 0.662)], intraocular pressures [17.5 ± 6.3 and 17.7 ± 6.5 (p = 0.894)], and use of glaucoma medication [1.4 ± 0.9 and 1.7 ± 1.1 medications (p = 0.218)]. At the final consultation, there remained no significant difference between the retrobulbar and topical anesthesia groups in VA (p = 0.644), IOP (p = 0.060), medication usage (p = 0.214), or complications (p = 0.580). Mean changes were also not statistically different among groups: VA (p = 0.910), IOP (p = 0.268), and medications (p = 0.963). Similar fluctuations in IOP, VA, and medication usage over time were seen, as shown in Graph 5. However, 1 week after surgery 15.6% of retrobulbar cases had a visual acuity depressed by more than 5 lines, while only 5.5% of topical cases observed the same decrease. By the final consultation, VA loss of greater than 5 lines had recovered to 3.1 and 1.8%, respectively. Log-rank comparison of Kaplan-Meier survival rates revealed a significantly greater survival rate for retrobulbar patients over topical patients under the criteria of complete success (p = 0.006), but no significant difference with qualified success criteria (p = 0.769).

## DISCUSSION

This retrospective review of phacotrabeculectomy cases found the two-site surgery with use of mitomycin C to be a safe and an effective procedure for glaucoma patients with cataracts in the Dominican Republic. Overall qualified and complete success after the first year was 95 and 76%, respectively. Topical anesthesia proved a viable replacement for retrobulbar anesthesia, achieving equivalent long-term results and without the risk of potential optic nerve complications.

Intraocular pressure can spike dangerously immediately after cataract surgery in open angle glaucoma eyes.^6^ Combined cataract-glaucoma surgery has been found to ameliorate pressure volatility.^19^ One day postoperatively, mean IOP was 13.7 mm Hg; however, 15% of patients were >21 mm Hg, a standard deviation of 8.3 mm Hg. Careful observation is critical in the days immediately following surgery. No patients received glaucoma medication during the first week, but by retraction of sutures and eye massages only 3% of patients were > 21 mm Hg after 1 week. Average patient visual acuity had returned to preoperative levels 1 week after surgery. At the final consultation (19.0 ± 12.2 months), visual acuity improved or remained unchanged in 90% of cases.

Mitomycin C application during phacotrabeculectomy surgery can reduce IOP an additional 2 to 4 mm Hg and is recommended for patients with risk factors for filtration failure, such as black race.^20,21^ However, it is also associated with late-term bleb leak and complications of blepharitis, endophthalmitis, and hypotony.^13,20^ Without MMC, Stark et al achieved a 78% medication-free rate over a period of 42 months in a primarily white population.^9^ We achieved a medication-free rate of 61% at the final consultation, and the average patient decreased 1.0 ± 1.3 medications. Our patients were predominantly of West Indian race and we found that with MMC application many patients were able to achieve target pressures without medication. There was a low overall risk of hypotony and a single case of blepharitis. Although we found strong results with MMC, our study did not directly monitor bleb leak nor compare MMC cases to non-MMC cases and we cannot evaluate the true necessity of MMC in West Indian patients.

The goal of our study was to compare topical and retrobulbar anesthesia in phacotrabeculectomy. Topical anesthesia has been used for many years in cataract and glaucoma surgeries.^16,22^ In combined phaco-trabeculectomy, as in separate surgeries, surgeons and patients report being as comfortable under topical as under retrobulbar anesthesia, achieving equivalent results.^14-17^ Our surgeon switched from retrobulbar to topical anesthesia in 2003. Patients from both anesthesia groups had statistically equivalent preoperative and postoperative variables of IOP, VA, and medication usage. Additionally, both groups had equivalent complication rates and qualified success rates (p = 0.769). Although the retrobulbar group had a higher complete success rate (p = 0.006), this may reflect the longer follow-up of the retrobulbar group and not a difference in complication rate. Complete success required no use of glaucoma medications, and as it is a propensity of patients to wean themselves off of medications, the lengthier follow-up of the retrobulbar group may be the differential factor for complete success.

A benefit of topical anesthesia that does not appear in the statistics of a study of our size is the removal of the risk of serious complications from the anesthesia injection. Glaucoma patients are predisposed to optic nerve complications, which although infrequent are devastating. We observed one ‘snuff-out’, a retrobulbar patient who fell from counting fingers to light perception, that might not have occurred under topical anesthesia.

Overall, our findings strongly support the several literature reviews that found two-site phacotrabeculectomy with MMC to be a safe and effective treatment for glaucoma patients with cataracts.^7,20,23^ Additionally, topical anesthesia proved to be a safe and successful alternative to retrobulbar anesthesia.

## References

[1] Veldman E, Greve EL (1987). Glaucoma filtering surgery, a retrospective study of 300 operations.. Doc Ophthalmol.

[2] Wishart PK, Austin MW (1993). Combined cataract extraction and trabeculectomy: phacoemulsification compared with extracapsular technique.. Ophthalmic Surg.

[3] Casson RJ, Salmon JF (2001). Combined surgery in the treatment of patients with cataract and primary open-angle glaucoma.. J Cataract Refract Surg.

[4] Jamarillo J Efectividad de la Facotrabeculectomía en patientes con cataratas y glaucoma.. Presented at Vision 2020 conference. Baranquilla, Columbia, 2006.

[5] Friedman DS, Jampel HD, Lubomski LH (2002). Surgical strategies for coexisting glaucoma and cataract: an evidence-based update.. Ophthalmology.

[6] Pohjalainen T, Vesti E, Uusitalo R, Laatikainen L (2001). Phacoemulsification and intraocular lens implantation in eyes with open-angle glaucoma.. Acta Ophthalmol Scand.

[7] Verges C, Cazal J, Lavin C (2005). Surgical strategies in patients with cataract and glaucoma.. Curr Opin Ophthalmol.

[8] Murthy SK, Damji KF, Pan Y, Hodge WG (2006). Trabeculectomy and phacotrabeculectomy, with mitomycin-C, show similar 2-year target IOP outcomes.. Can J Ophthalmol.

[9] Stark WJ, Goyal RK, Awad O (2006). The safety and efficacy of combined phacoemulsification and trabeculectomy with releasable sutures.. Br J Ophthalmol.

[10] Shingleton BJ, Price RS, O’Donoghue MW, Goyal S (2006). Comparison of 1-site versus 2-site phacotrabeculectomy.. J Cataract Refract Surg.

[11] Cagini C, Murdolo P, Gallai R (2003). Long-term results of one-site phacotrabeculectomy.. Acta Ophthalmol Scand.

[12] Shin DH, Hughes BA, Song MS (1996). Primary glaucoma triple procedure with or without adjunctive mitomycin. Prognostic factors for filtration failure.. Ophthalmol.

[13] DeBry PW, Perkins TW, Heatley G (2002). Incidence of late-onset bleb-related complications following trabeculectomy with mitomycin.. Arch Ophthalmol.

[14] Lai JS, Tham CC, Lam DS (2002). Topical anesthesia in phacotrabeculectomy.. J Glaucoma.

[15] Ahmed II, Zabriskie NA, Crandall AS (2002). Topical versus retrobulbar anesthesia for combined phacotrabeculectomy: prospective randomized study.. J Cataract Refract Surg.

[16] Zabriskie NA, Ahmed II, Crandall AS (2002). A comparison of topical and retrobulbar anesthesia for trabeculectomy.. J Glaucoma.

[17] Pablo LE, Ferreras A, Perez-Olivan S (2004). Contact-topical plus intracameral lidocaine versus peribulbar anesthesia in combined surgery: a randomized clinical trial.. J Glaucoma.

[18] Lai JS, Tham CC, Lam DS (2002). Topical anesthesia in phacotrabeculectomy.. J Glaucoma.

[19] Krupin T, Feitl ME, Bishop KI (1989). Postoperative intraocular pressure rise in open-angle glaucoma patients after cataract or combined cataract-filtration surgery.. Ophthalmol.

[20] Jampel HD, Friedman DS, Lubomski LH (2002). Effect of technique on intraocular pressure after combined cataract and glaucoma surgery: an evidence-based review.. Ophthalmol.

[21] Shin DH, Kim YY, Sheth N (1998). The role of adjunctive mitomycin C in secondary glaucoma triple procedure as compared to primary glaucoma triple procedure.. Ophthalmol.

[22] Patel BC, Burns TA, Crandall A (1996). A comparison of topical and retrobulbar anesthesia for cataract surgery.. Ophthalmol.

[23] Vass C, Menapace R (2004). Surgical strategies in patients with combined cataract and glaucoma.. Curr Opin Ophthalmol.

